# An Electrophysiological Study on the Neural Responses of Speaker Discrimination

**DOI:** 10.3390/bs16061011

**Published:** 2026-06-16

**Authors:** Puyang Geng, Xingui Wang, Hong Guo, Weibei Dou

**Affiliations:** 1 Department of Audio, Video, and Electronic Forensics, Academy of Forensic Science, Shanghai 200063, China; 2 Department of Electronic Engineering, Tsinghua University, Beijing 100084, China; wang-xg24@mails.tsinghua.edu.cn (X.W.); douwb@tsinghua.edu.cn (W.D.)

**Keywords:** speaker discrimination, event-related potentials, voice processing

## Abstract

The ability to distinguish speakers based on speech signals is a fundamental human ability essential for social communication, yet the neural mechanisms underlying this process remain poorly understood. The present study investigated the temporal dynamics of neural activity during speaker discrimination using event-related potentials (ERPs). Twenty-four native Mandarin speakers completed two tasks: an oddball session, in which participants passively listened to speech stimuli from standard and deviant speakers, and a voice line-up session, in which participants explicitly judged whether two consecutively presented speech stimuli were produced by the same or different speakers. In the oddball session, deviant stimuli elicited robust mismatch negativity (MMN) and P3a components compared to standard stimuli, indicating pre-attentive detection of speaker changes. In the voice line-up session, the different-speaker condition elicited more negative N1 and N400 amplitudes and more positive P2 amplitudes than the same-speaker condition, suggesting that speaker discrimination engages both early sensory processing and later cognitive integration. No significant differences were observed between the P300 and P600 components. These findings reveal distinct neural signatures associated with speaker-related processing across multiple temporal stages, with the MMN and P3a reflecting automatic detection of speaker-related acoustic changes, and the N1, P2, and N400 reflecting explicit speaker discrimination processes. While the present paradigm cannot fully isolate identity-level representations from low-level acoustic discrimination, the results provide novel ERP evidence on the temporal architecture engaged when listeners process speaker-specific information, contributing to a deeper understanding of speaker-related processing in the broader context of speaker identification research.

## 1. Introduction

The ability to distinguish speakers based on speech signals is a foundational human skill in social interactions. During social verbal communication, humans can rapidly extract speaker-related information, such as gender and age, and form vocal impressions to aid in speaker identification (von Kriegstein et al., 2005; Lavan et al., 2024; Park et al., 2025). This ability supports effective social interaction. While most studies have explored the auditory behavioral patterns associated with speaker identity (e.g., Best et al., 2023; Colby & Orena, 2022; Dougherty et al., 2015; McLaughlin et al., 2019), the understanding of the underlying neural mechanisms of speaker identity remains limited.

A growing body of electrophysiological research has focused on delineating the temporal dynamics of auditory processing, particularly through techniques such as fMRI, electroencephalography (EEG), and event-related potentials (ERPs). These methods allow researchers to capture brain responses on the millisecond scale, thereby revealing the rapid neural processes that underlie speech perception. However, neurological research on speaker identification remains profoundly insufficient, with a limited understanding of its neural basis.

### 1.1. Functional Organization in Cortical Speaker Identity Processing

A voice recognition model has been previously proposed where voice acts as an auditory “face”, conveying both linguistic and paralinguistic cues (e.g., emotions) (Belin et al., 2004). It has been suggested that voice information is initially processed via analyzing acoustic features in neural regions including the subcortical nuclei, Sylvian fissure, and superior temporal sulcus (STS). Existing neuroimaging studies (mainly via fMRI) have also shown that temporal voice areas (TVA, bilateral superior temporal gyri/sulci) are crucial for voice processing, with anterior and posterior STS (within TVA), and right precuneus activated during speaker identification tasks (Park et al., 2025; Scott, 2022; von Kriegstein et al., 2003). Bilateral STS, anterior temporal poles, and left amygdala also demonstrate sensitivity to speaker identity (Andics et al., 2010; Bestelmeyer & Mühl, 2022).

However, conflicting evidence suggests that TVA may not engage in high-order speaker identity processing when acoustic variations are controlled, with right posterior inferior frontal gyrus/primary motor cortex and left cingulate gyrus instead showing task sensitivity (Aglieri et al., 2021; Latinus et al., 2013). Recent studies have also emphasized the role of extra-temporal regions (e.g., inferior frontal gyrus, anterior insula) beyond the core network in speaker identification (Aglieri et al., 2021; McGettigan, 2015). Similarly, another study has found that brain activation patterns during speaker identification vary by language. For instance, recognizing Chinese-speaking voices, as opposed to English- or Ewe-speaking voices, is associated with reduced activity in the inferior frontal gyrus, precentral/postcentral gyri, supramarginal gyrus, and superior temporal sulcus/gyrus, while no significant differences were observed between English and Ewe voices (Meng et al., 2024).

### 1.2. Temporally Dynamic Responses of Speaker Identification

Apart from the functional organization of speaker identity processing, ERP studies using EEG for speaker identification remain scarce, with most existing research focusing on voice categorization (e.g., human vs. non-human, familiar vs. unfamiliar, male vs. female).

Previous studies have confirmed that the P1-N1-P2 complex is associated with sound detection, reflecting the neural encoding of temporal and spectral information (referred to in some studies as acoustic information) of sounds by the auditory cortex (Han, 2010; Martin et al., 2008; Park et al., 2025). As an early component of auditory processing, the P1-N1-P2 complex emerges sequentially after stimulus onset, within 50–200 ms, and is regarded as reflecting early sound detection (Martin et al., 2008; Wagner et al., 2017).

Though direct evidence regarding speaker identification remains limited, relevant studies have confirmed that acoustic changes in speech stimuli elicit the P1-N1-P2 complex (Han, 2010). Similarly, De Lucia et al. (2010) have found that when listening to human versus animal vocalizations, the early components (169–219 ms post-stimulus onset) were significantly stronger yet topographically indistinguishable, and localized to regions of the right superior temporal sulcus (STS) and superior temporal gyrus (STG). Sex identification elicits early N1/P2 modulation, indicating initial-stage processing stages (Latinus & Taylor, 2012; Park et al., 2025). Previous research on the auditory processing of speech in familiar versus unfamiliar speakers has similarly found that voices of familiar speakers evoke a more pronounced P2 component (Ma et al., 2023; Plante-Hébert et al., 2021).

The mismatch negativity (MMN) is another important event-related potential for studying auditory processing (Sussman et al., 2014). MMN has been widely used to investigate the pre-attentive processing and storage of regularities; when these regularities are violated, MMN is elicited (May & Tiitinen, 2010; Paavilainen, 2013). Thus, it is also used to study auditory change detection and can be triggered not only by pure tones but also by complex stimuli (e.g., speech) (Schwade et al., 2017; Zora et al., 2015).

Previous studies have found that the MMN is insensitive to changes in fundamental frequency (F0), which is a non-linguistic speaker identity cue (Jacobsen et al., 2004). They also found that the MMN is sensitive to changes in the first and second formants, which represent linguistic differences. However, Tuninetti et al. (2017) has found that accent and sex deviants yielded a larger MMN response compared to speaker and vowel deviants. This suggests that listeners automatically abstract F0 information and show a stronger response to stimuli with a larger F0 difference from the standard. Using an auditory oddball paradigm, studies comparing familiar and unfamiliar speakers have found that deviant stimuli elicit the MMN (Graux et al., 2015; Ma et al., 2023; Zinke et al., 2018), with the MMN showing greater amplitude for familiar speakers (Beauchemin et al., 2006; Graux et al., 2015). Piatti et al. (2021) also reported that young ASD (autism spectrum disorder) children without developmental delay exhibited greater MMN amplitude in processing deviant tones. Other researchers have reported conflicting findings that during syllable perception, the MMN exhibits reduced amplitude for trained voices compared to pre-training voices (Di Dona et al., 2021). Similarly, Ruiz-Martínez et al. (2020) found that children with ASD exhibited decreased MMN amplitudes when perceiving human sounds (used as deviants) compared to electronic sounds (used as standards).

Other later ERP components (e.g., P3, N400, P600, and LPC) have been associated with the detection of auditory oddities and reflect the neural processing of sound changes (Lim et al., 2021; Sussman et al., 2014). Some studies have reported significant P3a (Graux et al., 2015; Zinke et al., 2018) or LPC (Plante-Hébert et al., 2021) components for unfamiliar voices, while others find no significant ERP component (Föcker et al., 2011; Schall et al., 2015). Similarly, Conde et al. (2018) found that words spoken by participants themselves elicited increased P3 amplitudes relative to words spoken by unfamiliar speakers, suggesting that familiar voices engage greater attentional resources. Additionally, it has been proposed that voice identity influences linguistic processing, with identity changes eliciting significant N400 and P600 components (Ma et al., 2023; Xu et al., 2022). The LPC is generally associated with higher-order cognitive evaluation, and relevant studies have found that female voices elicit greater positive amplitude of the LPC than male voices, which is consistent with the listener-specific integration of speaker characteristics (Park et al., 2025).

### 1.3. Present Study

Before presenting our specific hypotheses, it is important to clarify the terminology used throughout this manuscript, as related but distinct constructs have sometimes been used interchangeably in the literature (Schweinberger et al., 2014). Following Schweinberger et al. (2014), we distinguish four levels of speaker-related processing: (a) voice categorization refers to the classification of a voice into broad categories such as gender or age; (b) speaker discrimination refers to the ability to judge whether two voices belong to the same or different speakers, without necessarily accessing identity-level representations; (c) speaker identification refers to the explicit recognition of a specific speaker’s identity, typically requiring access to stored representations of that individual; and (d) speaker recognition is often used as an umbrella term encompassing both discrimination and identification. We acknowledge that the present paradigm primarily engages speaker-related acoustic change detection (oddball) and speaker discrimination (voice line-up), and may not fully isolate identity-level representations.

The present study employed two complementary paradigms, viz., a passive oddball task and a voice line-up task, both of which primarily engage speaker discrimination rather than identity-level identification per se, because participants were unfamiliar with the speakers and were required only to detect or judge between-voice differences rather than to retrieve specific identities from long-term memory. Throughout this manuscript, we therefore use “speaker discrimination” when referring to the cognitive operations engaged by the present paradigm, and “speaker identification” when referring to the broader research domain or to prior studies that explicitly employed identification paradigms (e.g., involving familiar speakers or training-induced familiarity).

From the above literature review, it is clear that previous research has mainly focused on broad voice categorization (e.g., human vs. non-human, familiar vs. unfamiliar, male vs. female) rather than the more nuanced process of speaker identification. Perception of information such as speaker familiarity, gender, and accent all contribute to the extraction of speaker characteristic information, which is critical for achieving accurate speaker identification. However, findings regarding ERP components (e.g., MMN, P1) from prior studies on voice categorization remain subject to conflicting interpretations. Additionally, research specifically exploring the neural mechanisms underlying speaker identification remains very limited. To address the existing gap in ERP research on speaker-related processing and deepen insights into the neural mechanisms underpinning speaker discrimination of unfamiliar voices, this study seeks to delineate the temporal dynamics of neural activity during speaker discrimination tasks. It focuses on key ERP components (e.g., MMN, P1, N2, P300, etc.) associated with distinct stages of auditory processing.

Based on the multi-stage voice perception model (Belin et al., 2004), we formulated four targeted hypotheses corresponding to specific ERP components:

**H1.**  *For the oddball session, we predicted that deviant-speaker stimuli would elicit a larger MMN (100–250 ms) and P3a (250–400 ms) than standard stimuli, reflecting pre-attentive detection of speaker-related acoustic changes and involuntary attentional orienting, respectively.*

**H2.**  *For the voice line-up session, we predicted that the different-speaker condition would elicit larger N1 and P2 amplitudes (100–250 ms) than the same-speaker condition, reflecting enhanced early sensory encoding of acoustic deviation between successive speakers.*

**H3.**  *We predicted that the different-speaker condition would elicit a more negative N400 (400–600 ms), indexing context-based expectancy violation when the second voice mismatches the speaker established by the first.*

**H4.**  *For the late positive components (P300, P600), if explicit identification recruits additional decision-related and re-evaluation processes, condition differences may emerge; alternatively, comparable engagement across conditions would yield no significant modulations.*

## 2. Methods

### 2.1. Participants

This study obtained ethical approval from the Committee for the Protection of Human Subjects (CPHS) affiliated with the Academy of Forensic Science, Shanghai, China [No. 2023-15]. Twenty-four participants (seventeen males and seven females), aged between 22 and 36 years (M = 26.17, SD = 3.56), took part in the experiment. All were native Mandarin speakers, reported no history of auditory or neurological impairments, and were right-handed.

All participants were fully apprised of the research objectives, voluntarily signed a written informed consent form, and received financial remuneration upon the successful completion of the experimental procedures. It was also clearly communicated to the participants that they retained the right to withdraw from the study at any stage of the experiment.

### 2.2. Stimuli

All speech stimuli were collected from sixteen native speakers of Mandarin Chinese (eight male and eight female) aged from 24 to 40 years. The speakers were instructed to read /ma3/, /mi3/, and /mu3/ in their habitual speaking style. The speech stimuli were recorded using a SONY PCM-D100 recorder (manufactured by Sony [China] Co., Ltd. and sourced from Dongguan, China) at a sampling rate of 44.1 kHz and 16 bit resolution. All stimuli were RMS-normalized to a uniform intensity level of 70 dB SPL using Praat software (version 6.4.06). Stimulus durations were not normalized in order to preserve natural speaker-specific timing characteristics; the resulting duration distribution is reported in Table 1. No additional acoustic matching procedures were performed across speakers, so as to retain naturally occurring inter-speaker variability that defines speaker identity. These high-quality speech stimuli were then used to investigate the neural mechanism of speaker discrimination. Altogether 16 speakers × 3 = 48 speech stimuli were included in the perception experiment.

Acoustic analyses were conducted on all 48 speech stimuli using the Praat software (Boersma & Weenink, 2021). The extracted parameters included fundamental frequency (F0 mean, SD, and range), formant frequencies (F1, F2, F3), duration, harmonics-to-noise ratio (HNR), jitter, shimmer, and spectral tilt (H1−H2). As shown in Table 1, acoustic parameters exhibited expected sex-related differences. Female speakers showed significantly higher F0 mean (U = 572, *p* < 0.001), F0 SD (U = 483, *p* < 0.001), F0 range (U = 471, *p* < 0.001), and F3 (t = 5.23, *p* < 0.001) compared to male speakers. Male speakers exhibited significantly higher F1 (t = −3.22, *p* = 0.003) and longer stimulus durations (t = −2.87, *p* = 0.006). Spectral tilt differed significantly between sexes (U = 169, *p* = 0.015), and jitter was significantly higher for female speakers (U = 402, *p* = 0.019). No significant sex differences were observed for F2 (*p* = 0.063), HNR (*p* = 0.439), or shimmer (*p* = 0.439). These acoustic characteristics confirm the variability in speaker-specific voice parameters across the stimuli.

### 2.3. ERP Design

Building upon prior research, the present study designed two experimental sessions to comprehensively investigate the ERP components elicited during speaker discrimination, namely the Oddball session and the Voice Line-up session.

Oddball session. An oddball paradigm was employed in this session. The standard stimulus, consisting of the syllable /ma3/ produced by one speaker, was presented 300 times (probability of occurrence: *p* = 0.87), whereas the deviant stimulus, consisting of the same syllable /ma3/ produced by a different speaker, was presented 45 times (probability of occurrence: *p* = 0.13).

Voice line-up session. The voice line-up is a commonly employed paradigm in forensic speaker identification for determining speaker identity, wherein two voice samples are presented sequentially, and listeners/forensic experts are required to judge whether the two samples originate from the same speaker. This session comprised two sets, each containing 96 trials. For each set, 48 syllables produced by 16 speakers were randomly combined to form voice pairs from either the same speaker or different speakers, with 48 trials for each condition. Within each pair, the two stimuli always shared the same phonetic content (i.e., the identical target syllable, /ma3/, /mi3/, or /mu3/), so that same- and different-speaker pairs differed only in speaker identity and not in phonetic form. Different-speaker pairs were formed exclusively within the same gender (i.e., male–male or female–female pairings) rather than across genders, in order to prevent participants from relying on the highly salient gender cue when discriminating speakers and to ensure that judgments were based on finer speaker-specific acoustic characteristics. Gender was balanced across the same- and different-speaker conditions, with an equal number of male and female pairs in each condition.

### 2.4. Procedure

The perceptual experiment was conducted in a soundproof room. Participants sat in front of a laptop monitor (positioned for optimal viewing) and listened through high-quality in-ear headphones. The experiment was programmed using E-prime (Taylor & Marsh, 2017; version 3.0.3.80).

In the Oddball session, each trial began with a 600 ms display of a red fixation cross on a blank screen, instructing participants to maintain visual fixation while listening to the stimuli. Participants were also instructed to minimize large body movements, particularly those involving the head and hands.

In the Voice Line-up session, each trial started with a 400 ms red fixation cross, followed by two speech stimuli presented with a random inter-stimulus interval (ISI) ranging from 500 to 800 ms to minimize anticipation effects. After the presentation of both stimuli, a two-alternative forced-choice (2-AFC) task was administered, requiring participants to judge whether the two speech samples were produced by the same or different speakers. Participants responded using the keyboard, pressing “1” for same-speaker judgments and “0” for different-speaker judgments. Participants were allowed to rest as needed between the two sets before proceeding to the next set.

### 2.5. EEG Recording

**Figure 1:**
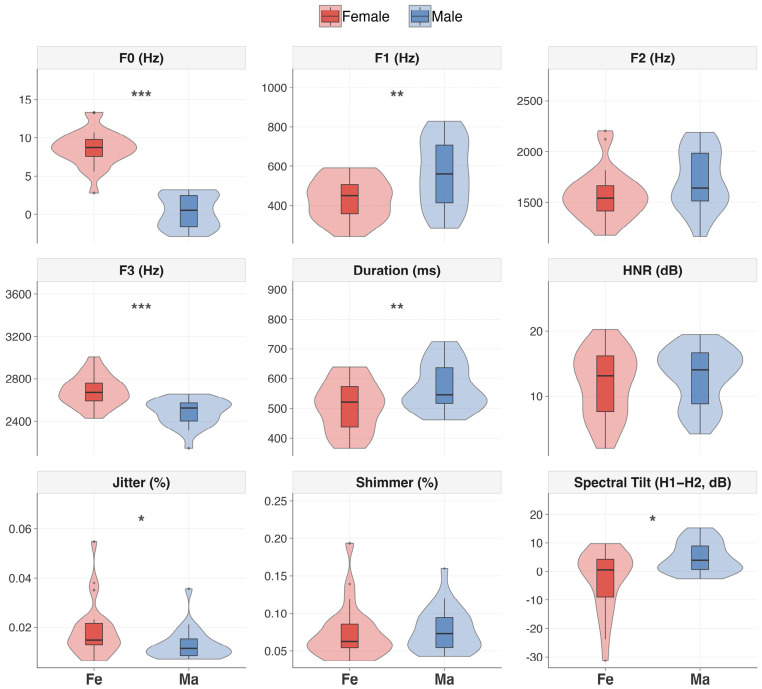
The acoustic statistics of all speech stimuli by gender. Asterisks indicate statistical significances (* *p* < 0.05, ** *p* < 0.01, *** *p* < 0.001).

**Figure 2:**
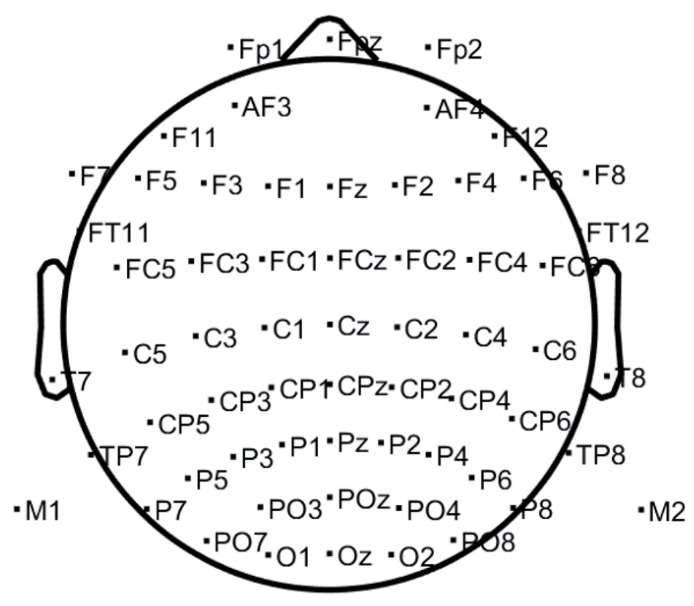
The spatial distribution of the 64 electrodes on the EEG cap.

EEG data were recorded using a Quick-Cap equipped with 64 sintered Ag/AgCl electrodes (see Figure 1 and Figure 2) connected to a Neuroscan SynAmps2 amplifier (Neuroscan Inc., Charlotte, NC, USA), with recordings managed using CURRY 8 software (Neuroscan Inc., Charlotte, NC, USA). Signals were acquired at a sampling rate of 1000 Hz. Two monopolar electrodes were placed on the left and right mastoids (M1 and M2), and two bipolar electrode pairs were used to record the vertical and horizontal electrooculogram (EOG) for monitoring eye movements and blinks. The electrode configuration followed the international 10–20 system, and electrode impedance was maintained below 10 kΩ.

### 2.6. EEG Analysis

Preprocessing and subsequent analyses of the EEG data were performed using CURRY 8 and the EEGLAB toolbox (version 2021.0). The preprocessing steps included re-referencing the signals and applying a band-pass filter between 1 and 40 Hz. The continuous data were then segmented into epochs spanning from −200 ms to 800 ms relative to stimulus onset, with a 200 ms pre-stimulus baseline correction applied. Independent component analysis (ICA) was subsequently performed to identify and remove artifacts associated with vertical and horizontal eye movements. Epochs containing amplitudes exceeding ±100 μV were excluded using the built-in automatic artifact rejection algorithm in EEGLAB.

Eighteen electrodes were selected for analysis. In the frontal region, nine electrodes were included: F3, FC3, and C3 in the left centro-frontal region; Fz, FCz, and Cz in the midline centro-frontal region; and F4, FC4, and C4 in the right centro-frontal region. In the posterior region, nine electrodes were included: CP3, P3, and PO3 in the left centro-posterior region; CPz, Pz, and POz in the midline centro-posterior region; and CP4, P4, and PO4 in the right centro-posterior region. The selection of these 18 fronto-central and centro-posterior electrodes was determined a priori, prior to inspection of the present data, based on prior ERP literature on voice processing (Föcker et al., 2011; Graux et al., 2015; Ma et al., 2023; Park et al., 2025; Plante-Hébert et al., 2021; Xu et al., 2022; Zinke et al., 2018), in which auditory ERP components (MMN, P3a, N1, P2, N400, P600) are typically reported to show maximal amplitude over fronto-central and centro-posterior scalp regions. Electrodes outside these a priori regions of interest were not included in the statistical models in order to constrain the family-wise error rate; this selection was therefore theory-driven rather than data-driven, and does not constitute circular analysis.

For the Oddball session, two time windows were defined: a mismatch negativity (MMN) window from 100 to 250 ms, with the MMN peak defined as the most negative amplitude within this interval, and a P3a window from 250 to 400 ms. For the Voice Line-up session, five time windows were defined: an N1 window from 100 to 150 ms, a P2 window from 150 to 250 ms, a P300 window from 250 to 400 ms, an N400 window from 400 to 600 ms, and a P600 window from 600 to 800 ms. Mean amplitudes, peak amplitudes, and peak latencies were measured across these eighteen electrodes.

Continuous EEG data were decomposed using independent component analysis (ICA) in EEGLAB. Independent components were classified using ICLabel, and those identified as ocular (vertical and horizontal eye movements), muscular, cardiac, line-noise, and other non-neural artifacts were removed prior to back-projection. On average, 17.67 components per participant (SD = 3.70, range: 11–26) were removed. Subsequent artifact rejection was performed using a ±100 μV amplitude threshold (EEGLAB pop_artextval). Across the 24 participants, 38,188 of 38,472 epochs (99.3%) were retained for analysis. The per-participant rejection rate was low (M = 0.7%, SD = 1.1%, range: 0.0–3.7%; retention 96.3–100%), indicating minimal artifact-related data loss well within accepted standards for ERP research.

For the Oddball session, no behavioral response was required, and therefore all artifact-free standard and deviant trials were included in the ERP averages. For the Voice Line-up session, ERP averages were computed from all artifact-free trials irrespective of whether the participant’s same/different judgment was correct. This decision was made to retain a sufficient and comparable number of trials per condition and to avoid confounding ERP amplitude estimates with behavioral accuracy; given the high overall accuracy (d′ = 1.35; see Section 3.2), the inclusion of a small proportion of incorrect trials is unlikely to have substantially affected the grand-average waveforms.

### 2.7. Data Analysis

Mean amplitudes, peak amplitudes, and peak latencies of the EEG measurements from the Oddball session (MMN and P3a) and the Voice Line-up session (N1, P2, P300, N400, and P600) at the eighteen electrodes were statistically analyzed using R software (R Core Team, 2020; version 4.5.1). Linear mixed-effects models (Bates et al., 2015) were employed to examine statistical differences in the EEG measurements. The EEG measurements served as dependent variables, while condition (Oddball session: standard vs. deviant; Voice Line-up session: same vs. different) and electrode were entered as fixed effects. Random intercepts by subject and random slopes for condition by subject were included in all models to support a maximal random effects structure (Barr et al., 2013). The degrees of freedom were estimated using the Satterthwaite approximation (Kuznetsova et al., 2017). The significance of the random slopes was evaluated using likelihood ratio tests, which confirmed that the slopes were significant across all model fittings. Tukey’s HSD post hoc tests were subsequently conducted for pairwise comparisons (Lenth, 2020). Partial eta-squared (η^2^) was reported as the measure of effect size with non-central 95% confidence intervals (Ben-Shachar et al., 2020).

Behavioral sensitivity was quantified using signal detection theory (Green & Swets, 1966). Same (repeated) trials were treated as signal and different trials as noise. For each participant, hit and false-alarm rates were computed and adjusted with the log-linear correction (Hautus, 1995; adding 0.5 to each frequency and 1 to each total) to accommodate extreme proportions. Sensitivity (d′) and response criterion (c) were then derived for each participant. d′ was tested against chance (zero) using a two-tailed one-sample t test; because a Shapiro–Wilk test indicated deviation from normality, a Wilcoxon signed-rank test was additionally conducted as a robustness check. Cohen’s d_z is reported as the effect size.

To explore the relationship between neural responses and behavioral performance, Pearson correlations were computed between ERP component amplitudes and task accuracy across participants. Correlation analyses were conducted for individual condition amplitudes (e.g., Same, Different, Standard, Deviant) as well as difference waves (D-waves: Different minus Same for the Voice Line-up session; Deviant minus Standard for the Oddball session).

## 3. Results

### 3.1. Oddball Session

**Figure 3:**
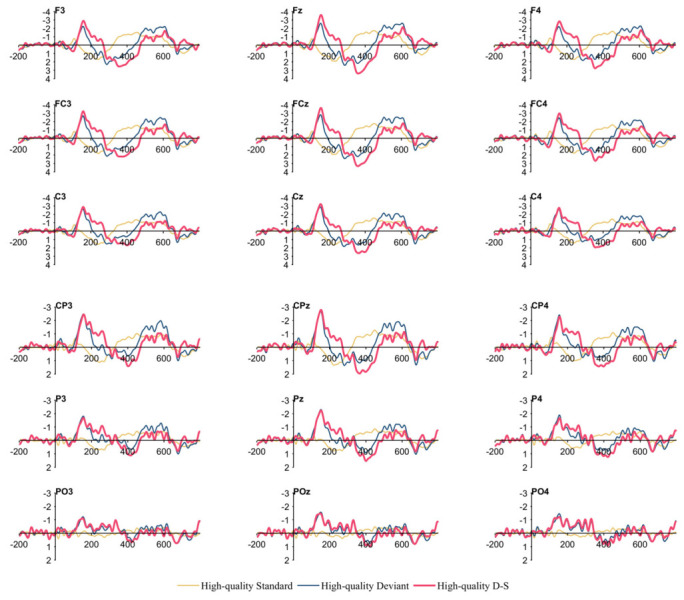
The grand average ERP waveforms for the Oddball session recorded at the eighteen electrodes. These waveforms include the ERP responses to both standard (yellow solid line) and deviant stimuli (blue solid line), as well as the difference waves obtained by subtracting the standard from the deviant (red solid line).

The grand average ERP waveforms for the Oddball session recorded at the eighteen electrodes are presented in Figure 3. These waveforms include the ERP responses to both standard and deviant stimuli, as well as the difference waves obtained by subtracting the standard from the deviant.

To identify significant MMN components, this study defined them as prominent negative deflections in the difference waves occurring approximately between 100 and 250 ms (Wang et al., 2024). A subsequent positive deflection, peaking around 250–400 ms, was used to identify the P3a component.

**Figure 4:**
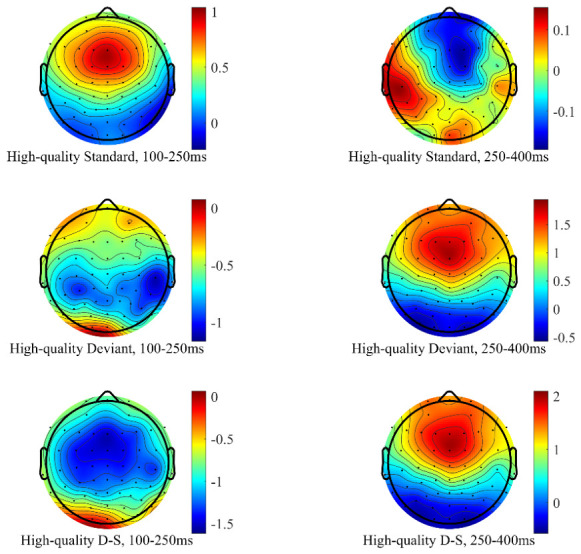
Brain topographic maps for standard and deviant stimuli, as well as the difference wave (deviant minus standard), in the 100–250 ms and 250–400 ms time windows.

As shown in Figure 4, topographic maps for the 100–250 ms and 250–400 ms time windows were analyzed to determine regional activation patterns. Visual inspection revealed a consistent frontal negativity for deviant stimuli in the 100–250 ms window, indicative of the MMN, and a frontal positivity for deviant stimuli in the 250–400 ms window, indicative of the P3a.

The results of the linear mixed-effects model analysis on EEG measurements from the Oddball session comparing standard and deviant stimuli at the 18 electrodes are presented in Table 2. For both the MMN and P3a components, significant main effects of “Condition” and “Electrode”, as well as significant two-way interaction effect of “Condition × Electrode”, were observed across all EEG measurements (*p* < 0.05).

As shown in Appendix A.1 and Appendix A.2, Tukey’s post hoc analysis revealed that for the MMN component, deviant stimuli elicited significantly more negative mean and peak amplitudes than standard stimuli across all 18 electrodes (*p* < 0.05). Additionally, deviant stimuli elicited significantly longer peak latencies than standard stimuli at all electrodes except those in the parieto-occipital region (i.e., PO3, PO4, and Poz) (*p* < 0.05). For the P3a component, deviant stimuli elicited significantly more positive peak amplitudes than standard stimuli across all 18 electrodes (*p* < 0.05), while this effect on mean amplitudes was observed only at fronto-central electrodes (i.e., C3, C4, Cz, F3, F4, FC3, FC4, FCz, Fz, and CPz) (*p* < 0.05). Additionally, deviant stimuli elicited significantly longer peak latencies than standard stimuli at all electrodes except those in the parieto-occipital region (i.e., PO3, PO4, POz, and Pz) (*p* < 0.05).

### 3.2. Voice Line-Up Session

Table 3 presents the accuracy and response times for participant judgments in the Voice Line-up session. Overall task accuracy (M = 77.6%, SD = 2.2%) was well above chance level (50%), indicating that participants successfully engaged in the speaker discrimination task. The low inter-subject variability in accuracy (SD = 2.2%, range: 72.9–85.4%) suggests consistent task performance across participants. Response times showed greater individual variability (M = 330.5 ms, SD = 132.3 ms, range: 197.7–669.6 ms). Based on a simple comparison of the mean values, accuracy and response times were similar between the same-speaker and different-speaker conditions.

Participants discriminated repeated from non-repeated stimuli well above chance. Sensitivity averaged d′ = 1.35 (SD = 0.17, range 0.84–1.50), and the response criterion was close to neutral (c = −0.12, SD = 0.21), indicating no substantial response bias. A one-sample t test confirmed that d′ was significantly greater than zero, t(23) = 38.01, *p* < 0.001, 95% CI [1.27, 1.42], Cohen’s d_z = 7.76. Because the d′ distribution deviated from normality (Shapiro–Wilk W = 0.70, *p* < 0.001), driven by two participants with comparatively low sensitivity (d′ ≈ 0.84), a Wilcoxon signed-rank test was also conducted and yielded the same conclusion, *p* < 0.001. Overall accuracy was 74.7% (SD = 3.9%), and the median reaction time on correct trials averaged 299 ms (SD = 82 ms). These results demonstrate that participants performed the task reliably and attentively.

**Figure 5:**
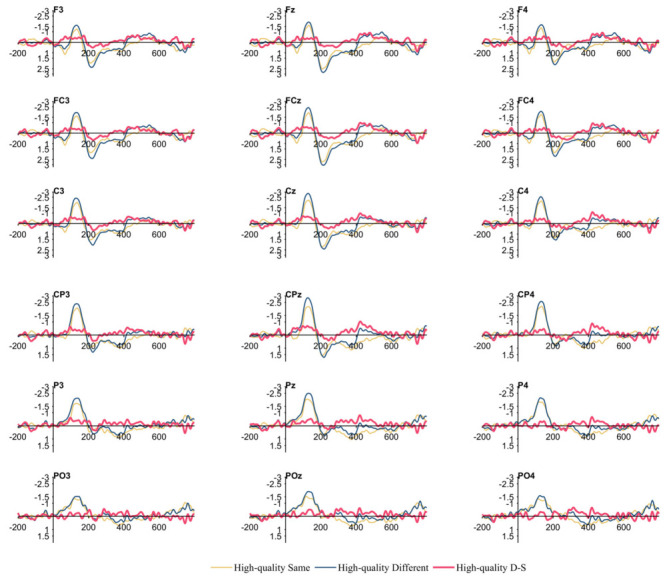
The grand average ERP waveforms for the Voice Line-up session recorded at the eighteen electrodes. These waveforms include the ERP responses to both the same-speaker condition (yellow solid line) and different-speaker condition (blue solid line), as well as the difference waves obtained by subtracting the same from the different (red solid line).

The grand average ERP waveforms for the Voice Line-up session recorded at the 18 electrodes are presented in Figure 5, including ERP responses to both same-speaker and different-speaker conditions, as well as difference waves (different minus same). Visual inspection of the waveforms revealed distinct N1-P2, P300, N400, and P600 components in both conditions at electrodes, with minimal differences observed between the same-speaker and different-speaker conditions.

**Figure 6:**
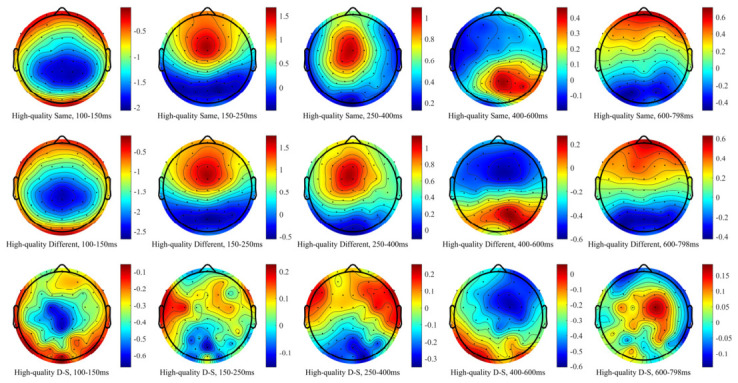
Brain topographic maps for same and different-speaker conditions, as well as the difference wave (different minus same), in the five time-windows.

As shown in Figure 6, topographic maps for the five time-windows (100–150 ms, 150–250 ms, 250–400 ms, 400–600 ms, and 600–800 ms) were analyzed to determine regional activation patterns. Visual inspection revealed a consistent parietal negativity (N1), frontal positivity (P2 and P300), fronto-parietal negativity (N400), and prefrontal positivity (P600). The topographic maps of the difference waves (different minus same) showed no discernible patterns.

The results of the linear mixed-effects model analysis on EEG measurements from the Voice Line-up session comparing same and different-speaker conditions at the 18 electrodes are presented in Appendix A.3. The results revealed significant main effects of “Condition” on mean and peak amplitudes for N1, peak amplitude for P2, and mean and peak amplitudes for N400 (*p* < 0.05). Significant main effects of “Electrode” were observed for all EEG measurements across all components (*p* < 0.05), except for the peak latency of N1 and P2. A significant “Condition × Electrode” interaction was found only for N1 peak latency (*p* < 0.05).

Tukey’s post hoc analysis on the significant main effects of “Condition” and the two-way interaction effects of “Condition × Electrode” revealed that the different-speaker condition elicited more negative mean amplitudes for N1 and N400 than the same-speaker condition (N1: Estimate = −0.39, SE = 0.17, t = −2.31, *p* = 0.03; N400: Estimate = −0.35, SE = 0.08, t = −4.15, *p* < 0.001). The different-speaker condition also elicited more negative peak amplitudes for N1 and N400, and more positive peak amplitudes for P2, compared to the same-speaker condition (N1: Estimate = −0.44, SE = 0.20, t = −2.26, *p* = 0.03; P2: Estimate = 0.39, SE = 0.15, t = 2.63, *p* = 0.02; N400: Estimate = −0.32, SE = 0.11, t = −2.88, *p* = 0.01). Additionally, the different-speaker condition elicited longer N1 peak latencies at Fz and P4 electrodes than the same-speaker condition (Fz: Estimate = 7.17, SE = 3.58, t = 2.00, *p* = 0.048; P4: Estimate = 8.42, SE = 3.58, t = 2.35, *p* = 0.02).

### 3.3. Brain-Behavior Correlations

To examine whether neural markers of speaker processing were associated with behavioral performance, Pearson correlations were computed between ERP amplitudes and task accuracy (N = 24). The results are summarized in Table 4.

**Figure 7:**
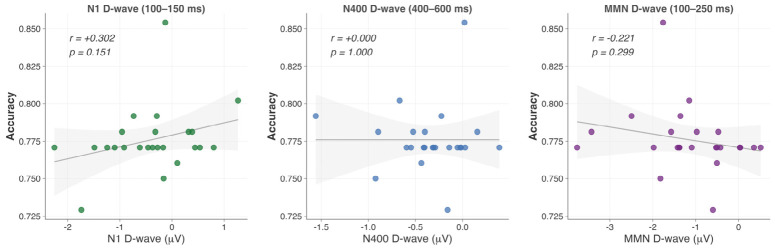
Correlations between behavioral performance (i.e., Accuracy) and ERP Difference waves (Different minus Same [Voice Line-up] or Deviant minus Standard [Oddball]).

No correlation reached statistical significance at α = 0.05. However, several trend-level associations were observed. As shown in Figure 7, for the N1 component, a trend-level negative correlation was found between same-speaker amplitude and accuracy (r = −0.394, *p* = 0.057), suggesting that participants who exhibited more negative (larger) N1 amplitudes for same-speaker trials tended to achieve higher accuracy. The N1 difference wave showed a positive trend with accuracy (r = 0.302, *p* = 0.151), indicating that larger neural differentiation between conditions (more negative for Different than Same) was weakly associated with better performance. For the MMN component, deviant amplitude showed a negative trend with accuracy (r = −0.308, *p* = 0.143), suggesting that stronger pre-attentive change detection (more negative MMN) was associated with better task performance. The N400 difference wave showed no association with accuracy (r ≈ 0.000, *p* > 0.999), indicating that although the N400 condition effect was robust at the group level (η^2^ = 0.43), it did not covary with individual differences in behavioral performance.

## 4. Discussion

The present study investigated the neural mechanisms underlying speaker discrimination by examining ERP components during an Oddball paradigm and a Voice Line-up task. Our findings revealed distinct temporal dynamics of neural processing associated with speaker-related processing, with significant modulations observed in both early and late ERP components.

### 4.1. Pre-Attentive Detection of Speaker Change: MMN and P3a

In the oddball session, deviant stimuli elicited robust MMN and P3a components compared to standard stimuli, indicating that listeners can pre-attentively discriminate between speakers. The MMN, typically observed between 100 and 250 ms post-stimulus onset, reflects the automatic detection of auditory regularities and their violations (May & Tiitinen, 2010; Paavilainen, 2013). Our finding that deviant speaker stimuli elicited significantly more negative MMN amplitudes across all 18 electrodes suggests that speaker-specific acoustic features are encoded in auditory memory and that deviations from the established speaker template trigger an automatic change-detection response.

This result extends previous research on voice categorization using the oddball paradigm. While Jacobsen et al. (2004) reported that MMN is insensitive to changes in fundamental frequency (F0), a non-linguistic speaker identity cue; our findings align more closely with Tuninetti et al. (2017), who found that speaker deviants yielded MMN responses, suggesting that listeners automatically abstract speaker-related acoustic information. The robust MMN observed in our study may reflect the integration of multiple acoustic cues beyond F0, such as formant structures and voice quality, which collectively contribute to speaker identity (Belin et al., 2004). Our results are also consistent with studies comparing familiar and unfamiliar speakers, which have demonstrated that deviant stimuli elicit MMN responses (Graux et al., 2015; Ma et al., 2023; Zinke et al., 2018).

The P3a component, observed between 250 and 400 ms, reflects the involuntary orienting of attention toward novel or salient stimuli (Sussman et al., 2014). Deviant stimuli in our study elicited significantly more positive P3a amplitudes, particularly at fronto-central electrodes, indicating that speaker changes captured attentional resources even in the absence of an explicit identification task. This finding is consistent with previous studies reporting significant P3a components for unfamiliar voices (Graux et al., 2015; Zinke et al., 2018) and suggests that speaker identity changes are sufficiently salient to trigger automatic attentional orienting. Notably, the fronto-central scalp distribution of the P3a effect observed in the current dataset only provides topographic ERP evidence at the scalp level. This surface distribution pattern cannot serve as definitive evidence to confirm the engagement of specific frontal neural regions (e.g., the inferior frontal gyrus) in speaker identity processing; any inference regarding underlying neural sources remains speculative without complementary source localization or neuroimaging data (Aglieri et al., 2021; McGettigan, 2015).

### 4.2. Neural Engagement During Speaker Discrimination

Examination of the grand average waveforms and topographic maps in the Voice Line-up session revealed prominent N1-P2, P300, N400, and P600 components during speaker discrimination judgment, indicating substantial neural resource allocation when listeners engaged in explicit speaker discrimination. These distinct ERP components suggest that speaker discrimination is a multi-stage process involving both early sensory analysis and later cognitive evaluation, consistent with the hierarchical model of voice processing proposed by previous studies (e.g., Belin et al. (2004) and Ohi et al. (2021)).

### 4.3. Early Sensory Processing of Speaker Identity: N1 and P2

Statistical analyses revealed significant modulations of the N1 and P2 components between the same-speaker and different-speaker conditions. For the N1 component (100–150 ms), the different-speaker condition elicited more negative amplitudes than the same-speaker condition, with a small-to-medium effect size (partial η^2^ = 0.19). For the P2 component (150–250 ms), the different-speaker condition elicited more positive peak amplitudes, also with a small-to-medium effect size (partial η^2^ = 0.23). The different-speaker condition elicited more negative N1 amplitudes and more positive P2 amplitudes compared to the same-speaker condition. The P1-N1-P2 complex is associated with early sound detection and reflects the neural encoding of temporal and spectral information by the auditory cortex (Han, 2010; Martin et al., 2008; Park et al., 2025). These early components, emerging within 50–200 ms post-stimulus onset, are regarded as markers of initial auditory feature extraction (Martin et al., 2008; Wagner et al., 2017).

Our N1 findings suggest that detecting a different speaker requires enhanced early sensory processing, possibly reflecting the increased neural effort needed to encode novel acoustic features that deviate from the previously heard speaker (Näätänen & Picton, 1987). These early effects align with the early-stage sensory component of speaker discrimination, in which acoustic feature extraction precedes any identity-level recognition. This interpretation is consistent with De Lucia et al. (2010), who found that early components (169–219 ms) were significantly modulated when listeners distinguished between human and animal vocalizations, with source localization implicating the right superior temporal sulcus and gyrus. Similarly, research on sex identification has demonstrated early N1/P2 modulation, indicating that speaker-related characteristics are processed at initial stages (Latinus & Taylor, 2012; Park et al., 2025; Schweinberger et al., 2014).

The enhanced P2 amplitude for different speaker trials aligns with previous findings that familiar speakers evoke more pronounced P2 components (Ma et al., 2023; Plante-Hébert et al., 2021). In our study, the increased P2 for different speakers may reflect heightened sensory processing demands when the acoustic input mismatches the listener’s expectation based on the preceding stimulus (Crowley & Colrain, 2004). The longer N1 peak latencies observed at Fz and P4 electrodes for different-speaker trials may suggest that processing speaker changes requires additional time for acoustic feature analysis. However, this effect should be interpreted with caution, given that it was observed at only two of the eighteen electrodes, indicating a highly localized and relatively weak effect. Whether this latency difference reflects a meaningful cognitive process or merely represents statistical noise warrants further investigation in future studies with larger samples and more diverse speaker stimuli.

### 4.4. Higher-Order Cognitive Processing: N400

A notable finding of the present study is the significant modulation of the N400 component (400–600 ms), with the different-speaker condition eliciting more negative N400 amplitudes than the same-speaker condition (partial η^2^ = 0.43 for mean amplitude, *p* < 0.001; partial η^2^ = 0.27 for peak amplitude, *p* = 0.008). This medium-to-large effect size indicates robust cognitive processing differences during speaker identity evaluation. The N400 is traditionally associated with semantic processing and the integration of meaningful information (Kutas & Federmeier, 2011; Lim et al., 2021). In contrast to the strong identity integration interpretation proposed in preliminary assumptions, the observed N400 modulation in the current speaker discrimination paradigm can be explained by multiple plausible cognitive mechanisms and should be interpreted conservatively. First, the enhanced N400 for different speaker voices may primarily index stimulus mismatch and contextual expectancy violation, rather than the specific integration of voice identity information with stored speaker template representations. During the sequential speaker judgment task, listeners form transient predictive expectations of upcoming vocal acoustic features based on preceding stimuli; a speaker change breaks this contextual expectation, triggering N400 enhancement. Second, while identity-related cognitive processing may contribute to this effect, the current ERP data cannot exclusively verify the involvement of speaker-specific representational integration. Multiple alternative explanations for the N400 effect remain valid, including general acoustic incongruency detection and non-semantic stimulus deviation processing (Belin et al., 2011). It should be noted that, because participants in our study were unfamiliar with the speakers, the N400 effect observed here likely reflects discrimination-related comparison processes rather than identity-level retrieval from long-term memory; whether the same N400 mechanism generalizes to identity-level speaker identification (e.g., with familiar speakers) remains an open question for future research.

This finding is consistent with previous research proposing that voice identity influences linguistic processing, with identity changes eliciting significant N400 components (Ma et al., 2023; Xu et al., 2022). Collectively, the current N400 results reliably demonstrate that speaker acoustic deviation modulates late cognitive ERP responses. However, we emphasize that these observations do not permit definitive conclusions regarding unique speaker representational processing. The N400 effect reported here aligns with domain-general functional characteristics of this component, which responds broadly to unexpected, incongruent, or mismatched sensory and contextual information across visual, auditory, and multisensory processing domains (Schweinberger & Burton, 2003; Stekelenburg & Vroomen, 2007; Yu et al., 2022). Thus, the present data support the moderate conclusion that speaker identity change constitutes a salient contextual violation that modulates late-stage cognitive processing, rather than confirming a dedicated neural mechanism for speaker representation integration.

### 4.5. Neural Engagement Without Condition-Specific Modulation: P300 and P600

Although we observed prominent P300 and P600 components in the grand average waveforms during the Voice Line-up task, no significant differences were found between the same-speaker and different-speaker conditions for these components. Importantly, the presence of robust P300 and P600 waveforms indicates that these components were actively engaged during speaker discrimination, reflecting attentional allocation and higher-order cognitive evaluation processes (Donchin & Coles, 1988; Polich, 2012). The absence of condition-specific modulation does not imply that these components are uninvolved in speaker discrimination; rather, it suggests that both same-speaker and different-speaker judgments recruited comparable levels of attentional and evaluative processing.

Previous studies have reported significant P3 components for speaker-related processing, with familiar voices eliciting increased P3 amplitudes (Beauchemin et al., 2006; Conde et al., 2018). The lack of P300 modulation between conditions in our study may be attributed to the nature of the task demands. Participants in the Voice Line-up session were required to make explicit same/different judgments (i.e., speaker discrimination judgments) for all trials, which likely engaged similar decision-related processes regardless of whether the speakers were the same or different (Verleger et al., 2005). This consistent engagement of attentional resources across both conditions may have obscured any condition-specific P300 effects.

Similarly, although the P600 and late positive complex (LPC) have been associated with higher-order cognitive evaluation of speaker characteristics (Park et al., 2025; Plante-Hébert et al., 2021) and re-analysis processes in language comprehension (Friederici, 2002), the absence of P600 differences between conditions may reflect the relatively straightforward nature of our voice line-up task. Participants made same/different judgments based on immediately preceding stimuli, which may have imposed equivalent evaluative demands across both conditions. Additionally, it is possible that once early sensory (N1, P2) and semantic integration (N400) processes had sufficiently extracted speaker identity information, both conditions required similar levels of higher-order evaluation for response execution, resulting in comparable P600 amplitudes (Kutas & Federmeier, 2011). Future studies employing more complex speaker identification paradigms or manipulating task difficulty may reveal condition-specific modulations in these later components. Crucially, we refrain from overinterpreting the null findings of P300 and P600 in the current study. The non-significant between-condition differences only indicate a lack of statistical modulation under the current task and stimulus parameters, and cannot be generalized to infer the absence of speaker-related functional roles of P300/P600 in voice processing. Furthermore, no definitive inferences regarding differential higher-order re-analysis or template-based speaker evaluation processes between conditions can be drawn based on these null ERP results.

### 4.6. Neural Substrates of Speaker Discrimination

Our ERP findings provide temporal evidence that complements existing neuroimaging research on the functional organization of speaker-related processing. The early effects observed in the N1 and P2 components are consistent with the involvement of temporal voice areas, including the superior temporal sulcus and gyrus, in initial acoustic analysis of speaker-related information (Belin et al., 2004; Park et al., 2025; Scott, 2022; von Kriegstein et al., 2003).

Consistent with the cautious interpretation principle for ERP data, the scalp topographic patterns of MMN and P3a effects observed in this study cannot provide rigorous evidence for the activation of specific extra-temporal neural regions (e.g., inferior frontal gyrus). ERP scalp distribution data alone lack the spatial resolution to pinpoint underlying neural sources, and all region-related functional hypotheses are tentative and require independent verification via fMRI, EEG source modeling, or other neuroimaging techniques (Aglieri et al., 2021; McGettigan, 2015).

In line with the above revisions, the current N400 modulation results reliably reflect multi-stage cognitive processing of speaker discrimination at the temporal level. However, without spatial neural evidence, we cannot draw definitive conclusions about the engagement of specific brain regions beyond temporal voice areas or confirm discrete neural substrate differences between sensory and cognitive speaker processing stages. The multi-stage processing model of speaker discrimination supported here is limited to temporal ERP dynamics, rather than spatial neural mechanisms. This finding supports the view that speaker discrimination—and, by extension, speaker identification in general—is a multi-stage process involving both automatic and controlled processing mechanisms (Belin et al., 2004; Ohi et al., 2021).

### 4.7. Brain-Behavior Relationships

An important question in speaker identification research more broadly concerns whether neural markers of speaker discrimination are predictive of behavioral performance. Our exploratory correlation analyses revealed several trend-level associations that, while not reaching statistical significance, provide preliminary insights into brain-behavior relationships in speaker discrimination.

For the N1 component, a trend-level negative correlation was observed between amplitude in the same-speaker condition and task accuracy (r = −0.394, *p* = 0.057). This suggests that participants who exhibited larger (more negative) N1 responses when processing same-speaker trials tended to perform better on the speaker discrimination task. Additionally, the N1 difference wave (Different minus Same) showed a positive trend with accuracy (r = 0.302, *p* = 0.151), indicating that participants with greater neural differentiation between speaker conditions at the early sensory processing stage tended to achieve higher accuracy. These patterns are consistent with the interpretation that enhanced early auditory processing supports more accurate speaker discrimination.

For the MMN component in the Oddball session, deviant amplitude showed a negative trend with accuracy (r = −0.308, *p* = 0.143), suggesting that participants with stronger pre-attentive detection of speaker changes (reflected in more negative MMN responses to deviant stimuli) tended to perform better on the explicit speaker discrimination task. This finding provides preliminary evidence that automatic change detection mechanisms indexed by the MMN may contribute to individual differences in speaker discrimination ability.

Interestingly, the N400 difference wave showed no association with accuracy (r ≈ 0.000, *p* > 0.999), despite the N400 showing the largest condition effect at the group level (η^2^ = 0.43). This dissociation suggests that while the N400 reliably indexes speaker identity processing at the cognitive integration stage, individual differences in N400 amplitude do not predict behavioral performance in the current sample. It is possible that once early sensory discrimination (N1) has successfully differentiated speaker identity, later cognitive processes (N400) operate relatively uniformly across individuals.

These correlation findings should be interpreted with caution, given that none reached conventional statistical significance (α = 0.05). The limited sample size (N = 24) likely constrained statistical power for detecting individual difference effects. Nevertheless, the consistent direction of effects for N1 (r = 0.302 for D-wave) and MMN (r = −0.221 for D-wave) supports the hypothesis that stronger neural differentiation between speaker conditions is associated with better behavioral discrimination ability. Future studies with larger samples are needed to confirm these preliminary brain-behavior associations.

### 4.8. Limitations

Several limitations of the present study should be acknowledged. First, the stimuli were produced by only 16 speakers (eight male and eight female), which may have limited acoustic variability across trials and contributed to the relatively subtle ERP differences observed in the voice line-up session. Second, the sample consisted solely of native Mandarin speakers (N = 24), which not only limits the generalizability of our findings, given that speaker-identification-related brain activation varies across languages (Meng et al., 2024), but also constrains the statistical power of individual-difference analyses. Indeed, exploratory brain–behavior correlations revealed consistent trend-level associations (e.g., N1 Same × Accuracy: r = −0.394, *p* = 0.057; N1 D-wave × Accuracy: r = 0.302, *p* = 0.151; MMN Deviant × Accuracy: r = −0.308, *p* = 0.143), but none reached statistical significance. Third, the voice line-up task required explicit same/different judgments, which may have introduced decision-related processes that obscured condition-specific effects in components such as the P300. Fourth, because all speakers were unfamiliar to the participants, our findings primarily speak to speaker discrimination—the ability to judge whether two voices belong to the same or different speakers—rather than to identity-level speaker identification, which typically requires retrieval of specific speaker identities from long-term memory. This conceptual scope also means that our results may not generalize to familiar-voice recognition, the neural mechanisms of which have been shown to differ substantially (Beauchemin et al., 2006; Graux et al., 2015).

Future research could address these limitations by incorporating more acoustically diverse speaker stimuli, recruiting larger and cross-linguistic samples together with tasks that elicit greater behavioral variability, and adopting paradigms that contrast familiar and unfamiliar voices or employ voice–identity training. Such efforts will help determine whether the neural signatures reported here generalize to genuine identity-level speaker identification.

Because speaker identity is inherently defined by a covarying bundle of acoustic features, the present design did not permit a clean dissociation of individual acoustic parameters, and we therefore did not model specific acoustic features as predictors of single-trial ERP amplitudes. Determining which acoustic dimensions drive the observed ERP modulations will require parametrically controlled or resynthesized voices in future work.

## 5. Conclusions

In summary, the present study reveals distinct neural signatures associated with speaker discrimination across multiple temporal stages in native Mandarin listeners. The robust MMN and P3a components observed in the oddball session indicate pre-attentive detection of speaker changes, while the N1, P2, and N400 modulations in the voice line-up session reflect both early sensory analysis and later cognitive integration during explicit speaker discrimination of unfamiliar voices. These findings contribute to a deeper understanding of the neural mechanisms underlying speaker-related processing, particularly speaker discrimination. Whether these neural signatures generalize to identity-level speaker identification—for instance, when listeners must retrieve familiar speakers from long-term memory—remains an important question for future research using familiar voices or training-induced familiarity paradigms. The present work thus provides a foundation for future research exploring the temporal dynamics of voice processing across the full spectrum from speaker discrimination to speaker identification.

## Figures and Tables

**Table 1:** Acoustic parameters by speaker gender (N = 48 stimuli).

Parameter	Female (N = 24)	Male (N = 24)	Test	*p*
F0 mean (Hz)	208.65 ± 23.33	120.37 ± 21.13	Mann–Whitney U	<0.001
F0 SD (Hz)	30.05 ± 16.50	15.30 ± 7.20	Mann–Whitney U	<0.001
F0 range (Hz)	95.55 ± 56.10	46.30 ± 22.60	Mann–Whitney U	<0.001
F1 mean (Hz)	432.59 ± 104.26	563.85 ± 170.17	Welch t	0.003
F2 mean (Hz)	1573.10 ± 243.69	1720.77 ± 290.38	Welch t	0.063
F3 mean (Hz)	2684.21 ± 142.30	2482.95 ± 123.61	Welch t	<0.001
Duration (ms)	506.05 ± 83.74	573.49 ± 79.05	Welch t	0.006
HNR (dB)	11.90 ± 5.12	13.05 ± 4.34	Mann–Whitney U	0.439
Jitter (%)	0.02 ± 0.01	0.01 ± 0.01	Mann–Whitney U	0.019
Shimmer (%)	0.07 ± 0.04	0.08 ± 0.03	Mann–Whitney U	0.439
Spectral Tilt (H1−H2, dB)	−2.80 ± 10.73	5.01 ± 5.29	Mann–Whitney U	0.015

**Table 2:** The results of LMM analysis on the mean amplitudes, peak amplitudes, and peak latencies of MMN and P3a components between standard and deviant stimuli at the eighteen electrodes.

Component	Measurements	Factor	df	F	*p*	η^2^	95%CI
MMN	Mean amplitudes	Condition	1	28.04	<0.001	0.55	[0.30, 1.00]
		Electrode	17	5.84	<0.001	0.11	[0.06, 1.00]
		Condition × Electrode	17	2.69	<0.001	0.06	[0.02, 1.00]
	Peak amplitudes	Condition	1	55.99	<0.001	0.71	[0.52, 1.00]
		Electrode	17	6.38	<0.001	0.12	[0.07, 1.00]
		Condition × Electrode	17	2.02	0.009	0.04	[0.01, 1.00]
	Peak latencies	Condition	1	23.52	<0.001	0.51	[0.25, 1.00]
		Electrode	17	5.55	<0.001	0.11	[0.06, 1.00]
		Condition × Electrode	17	2.17	0.004	0.05	[0.01, 1.00]
P3a	Mean amplitudes	Condition	1	14.15	0.001	0.38	[0.13, 1.00]
		Electrode	17	19.69	<0.001	0.30	[0.25, 1.00]
		Condition × Electrode	17	26.92	<0.001	0.37	[0.32, 100]
	Peak amplitudes	Condition	1	70.13	<0.001	0.75	[0.59, 1.00]
		Electrode	17	28.46	<0.001	0.38	[0.33, 1.00]
		Condition × Electrode	17	8.96	<0.001	0.16	[0.11, 1.00]
	Peak latencies	Condition	1	9.64	0.005	0.30	[0.07, 1.00]
		Electrode	17	2.41	0.001	0.05	[0.01, 1.00]
		Condition × Electrode	17	2.21	0.003	0.05	[0.01, 1.00]

**Table 3:** Descriptive statistics for accuracy and response times in the Voice Line-up session.

Condition	Measurement	Average Value	Range
All	Accuracy	77.6% ± 2.2%	[72.9%, 85.4%]
	Reaction time	330.5 ± 132.3 ms	[197.7, 669.6] ms
Same speaker	Accuracy	77.60%	-
	Reaction time	345.47 ms	-
Different speaker	Accuracy	73.44%	-
	Reaction time	392.51 ms	-

**Table 4:** Pearson correlations between ERP amplitudes and task accuracy.

Component	Contrast	r	*p*	95% CI
N1 (100–150 ms)	Same × Accuracy	−0.394	0.057	[−0.688, 0.011]
	Different × Accuracy	−0.156	0.465	[−0.527, 0.264]
N400 (400–600 ms)	D-wave × Accuracy	0.302	0.151	[−0.115, 0.629]
	Same × Accuracy	−0.160	0.455	[−0.529, 0.260]
MMN (100–250 ms)	Different × Accuracy	−0.205	0.336	[−0.562, 0.216]
	D-wave × Accuracy	0.000	>0.999	[−0.403, 0.404]
	Deviant × Accuracy	−0.308	0.143	[−0.633, 0.109]
	Standard × Accuracy	−0.157	0.464	[−0.527, 0.263]
	D-wave × Accuracy	−0.221	0.299	[−0.574, 0.200]

Note: D-wave = Different minus Same (Voice Line-up) or Deviant minus Standard (Oddball). Negative r values for amplitude × accuracy indicate that more negative amplitudes are associated with higher accuracy.

## Data Availability

The original contributions presented in this study are included as Supplementary Materials. Further inquiries can be directed to the corresponding authors.

## Supplementary Materials

The following supporting information can be downloaded at: https://www.mdpi.com/article/10.3390/bs16061011/s1.

## Appendix A

### Appendix A.1. Tukey’s HSD Post Hoc Analysis Results for the Two-Way Interaction Effect of “Condition × Electrode” on the MMN Component in the Oddball Session

**Table A1:** Tukey’s HSD post hoc analysis results for the two-way interaction effect of “Condition × Electrode” on the MMN component in the Oddball session using linear mixed-effects models.

Measurement	Electrode	β	SE	t	*p*
Mean amplitude	C3	−1.36	0.27	−5.03	<0.001
	C4	−1.26	0.27	−4.68	<0.001
	CP3	−1.29	0.27	−4.76	<0.001
	CP4	−1.10	0.27	−4.08	<0.001
	CPz	−1.24	0.27	−4.58	<0.001
	Cz	−1.36	0.27	−5.04	<0.001
	F3	−1.20	0.27	−4.43	<0.001
	F4	−1.27	0.27	−4.70	<0.001
	FC3	−1.41	0.27	−5.22	<0.001
	FC4	−1.25	0.27	−4.64	<0.001
	FCz	−1.48	0.27	−5.48	<0.001
	Fz	−1.52	0.27	−5.63	<0.001
	P3	−0.88	0.27	−3.27	0.002
	P4	−0.94	0.27	−3.47	<0.001
	PO3	−0.56	0.27	−2.07	0.04
	PO4	−0.76	0.27	−2.83	0.007
	POz	−0.76	0.27	−2.82	0.007
	Pz	−1.12	0.27	−4.15	<0.001
Peak amplitude	C3	−2.31	0.35	−6.68	<0.001
	C4	−2.16	0.35	−6.26	<0.001
	CP3	−2.16	0.35	−6.25	<0.001
	CP4	−1.85	0.35	−5.35	<0.001
	CPz	−2.35	0.35	−6.79	<0.001
	Cz	−2.44	0.35	−7.06	<0.001
	F3	−2.10	0.35	−6.08	<0.001
	F4	−2.08	0.35	−6.02	<0.001
	FC3	−2.41	0.35	−6.98	<0.001
	FC4	−2.20	0.35	−6.36	<0.001
	FCz	−2.49	0.35	−7.21	<0.001
	Fz	−2.48	0.35	−7.19	<0.001
	P3	−1.77	0.35	−5.12	<0.001
	P4	−1.93	0.35	−5.60	<0.001
	PO3	−1.44	0.35	−4.18	<0.001
	PO4	−1.82	0.35	−5.25	<0.001
	POz	−1.74	0.35	−5.04	<0.001
	Pz	−2.05	0.35	−5.94	<0.001
Peak latency	C3	20.75	8.35	2.49	0.01
	C4	22.75	8.35	2.72	0.007
	CP3	23.08	8.35	2.76	0.006
	CP4	35.33	8.35	4.23	<0.001
	CPz	31.58	8.35	3.78	<0.001
	Cz	24.33	8.35	2.91	0.004
	F3	28.67	8.35	3.43	0.001
	F4	30.25	8.35	3.62	<0.001
	FC3	18.50	8.35	2.22	0.03
	FC4	34.17	8.35	4.09	<0.001
	FCz	20.00	8.35	2.40	0.02
	Fz	20.58	8.35	2.47	0.01
	P3	20.25	8.35	2.43	0.02
	P4	25.17	8.35	3.01	0.003
	PO3	−1.75	8.35	−0.21	0.83
	PO4	2.17	8.35	0.26	0.80
	POz	0.00	8.35	0.00	1
	Pz	16.50	8.35	1.98	0.049

### Appendix A.2. Tukey’s HSD Post Hoc Analysis Results for the Two-Way Interaction Effect of “Condition × Electrode” on the P3a Component of Oddball Session

**Table A2:** Tukey’s HSD post hoc analysis results for the two-way interaction effect of “Condition × Electrode” on the P3a component in the Oddball session using linear mixed-effects models.

Measurement	Electrode	β	SE	t	*p*
Mean amplitude	C3	0.87	0.25	3.41	0.001
	C4	0.94	0.25	3.71	0.001
	CP3	0.28	0.25	1.11	0.27
	CP4	0.48	0.25	1.90	0.06
	CPz	0.94	0.25	3.71	0.001
	Cz	1.47	0.25	5.80	<0.001
	F3	1.59	0.25	6.25	<0.001
	F4	1.46	0.25	5.74	<0.001
	FC3	1.33	0.25	5.26	<0.001
	FC4	1.38	0.25	5.45	<0.001
	FCz	1.96	0.25	7.73	<0.001
	Fz	1.89	0.25	7.45	<0.001
	P3	−0.04	0.25	−0.14	0.89
	P4	0.17	0.25	0.66	0.51
	PO3	−0.35	0.25	−1.37	0.18
	PO4	−0.27	0.25	−1.07	0.29
	POz	−0.21	0.25	−0.82	0.42
	Pz	0.29	0.25	1.16	0.25
Peak amplitude	C3	1.65	0.33	5.06	<0.001
	C4	2.05	0.33	6.29	<0.001
	CP3	1.17	0.33	3.58	0.001
	CP4	1.58	0.33	4.85	<0.001
	CPz	1.81	0.33	5.55	<0.001
	Cz	2.44	0.33	7.50	<0.001
	F3	2.53	0.33	7.78	<0.001
	F4	2.52	0.33	7.73	<0.001
	FC3	2.08	0.33	6.39	<0.001
	FC4	2.36	0.33	7.25	<0.001
	FCz	3.14	0.33	9.64	<0.001
	Fz	2.94	0.33	9.02	<0.001
	P3	0.70	0.33	2.14	0.03
	P4	1.27	0.33	3.90	<0.001
	PO3	0.80	0.33	2.46	0.02
	PO4	0.92	0.33	2.82	0.006
	POz	1.02	0.33	3.13	0.002
	Pz	1.15	0.33	3.54	0.001
Peak latency	C3	42.67	12.90	3.32	0.002
	C4	37.42	12.90	2.91	0.005
	CP3	28.83	12.90	2.24	0.03
	CP4	35.50	12.90	2.76	0.008
	CPz	34.75	12.90	2.70	0.009
	Cz	26.25	12.90	2.04	0.046
	F3	36.25	12.90	2.82	0.007
	F4	39.67	12.90	3.09	0.003
	FC3	44.75	12.90	3.48	0.001
	FC4	38.83	12.90	3.02	0.004
	FCz	31.17	12.90	2.42	0.02
	Fz	54.42	12.90	4.23	<0.001
	P3	30.58	12.90	2.38	0.02
	P4	37.83	12.90	2.94	0.005
	PO3	13.33	12.90	1.04	0.30
	PO4	13.67	12.90	1.06	0.29
	POz	9.08	12.90	0.71	0.48
	Pz	24.67	12.90	1.92	0.06

### Appendix A.3. The Results of LMM Analysis on the Mean Amplitudes, Peak Amplitudes, and Peak Latencies of Five Components (i.e., N1, P2, P300, N400, P600) Between the Same and Different-Speaker Conditions

**Table A3:** The results of LMM analysis on the mean amplitudes, peak amplitudes, and peak latencies of five components (i.e., N1, P2, P300, N400, P600) between the same and different-speaker conditions at the eighteen electrodes.

Component	Measurements	Factor	df	F	*p*	η^2^	95%CI
N1	Mean amplitudes	Condition	1	5.34	0.03	0.19	[0.01, 1.00]
		Electrode	17	21.69	<0.001	0.32	[0.27, 1.00]
		Condition × Electrode	17	0.68	0.82	0.02	[0.00, 1.00]
	Peak amplitudes	Condition	1	5.10	0.03	0.18	[0.01, 1.00]
		Electrode	17	15.62	<0.001	0.25	[0.20, 1.00]
		Condition × Electrode	17	0.34	0.99	0.01	[0.00, 1.00]
	Peak latencies	Condition	1	0.21	0.65	0.01	[0.00, 1.00]
		Electrode	17	0.86	0.62	0.02	[0.00, 1.00]
		Condition × Electrode	17	1.95	0.01	0.04	[0.004, 1.00]
P2	Mean amplitudes	Condition	1	0.02	0.90	0.001	[0.00, 1.00]
		Electrode	17	53.71	<0.001	0.54	[0.50, 1.00]
		Condition × Electrode	17	0.16	1.00	0.004	[0.00, 1.00]
	Peak amplitudes	Condition	1	6.89	0.02	0.23	[0.03, 1.00]
		Electrode	17	53.85	<0.001	0.54	[0.50, 1.00]
		Condition × Electrode	17	0.57	0.91	0.01	[0.00, 1.00]
	Peak latencies	Condition	1	3.53	0.07	0.13	[0.00, 1.00]
		Electrode	17	1.51	0.08	0.03	[0.00, 1.00]
		Condition × Electrode	17	0.51	0.95	0.01	[0.00, 1.00]
P300	Mean amplitudes	Condition	1	0.23	0.64	0.01	[0.00, 1.00]
		Electrode	17	10.18	<0.001	0.18	[0.13, 1.00]
		Condition × Electrode	17	0.80	0.69	0.02	[0.00, 100]
	Peak amplitudes	Condition	1	0.00	0.99	0.00	[0.00, 1.00]
		Electrode	17	8.33	<0.001	0.15	[0.10, 1.00]
		Condition × Electrode	17	1.34	0.16	0.03	[0.00, 100]
	Peak latencies	Condition	1	1.88	0.18	0.08	[0.00, 1.00]
		Electrode	17	4.54	<0.001	0.09	[0.04, 1.00]
		Condition × Electrode	17	0.53	0.94	0.01	[0.00, 100]
N400	Mean amplitudes	Condition	1	13.21	<0.001	0.43	[0.17, 1.00]
		Electrode	17	13.46	<0.001	0.23	[0.17, 1.00]
		Condition × Electrode	17	1.36	0.15	0.03	[0.00, 100]
	Peak amplitudes	Condition	1	8.30	0.008	0.27	[0.05, 1.00]
		Electrode	17	11.25	<0.001	0.20	[0.14, 1.00]
		Condition × Electrode	17	0.78	0.72	0.02	[0.00, 100]
	Peak latencies	Condition	1	2.47	0.13	0.10	[0.00, 1.00]
		Electrode	17	2.31	0.002	0.05	[0.01, 1.00]
		Condition × Electrode	17	0.67	0.83	0.01	[0.00, 100]
P600	Mean amplitudes	Condition	1	0.43	0.52	0.02	[0.00, 1.00]
		Electrode	17	25.19	<0.001	0.35	[0.30, 1.00]
		Condition × Electrode	17	0.29	0.99	0.01	[0.00, 100]
	Peak amplitudes	Condition	1	0.49	0.49	0.02	[0.00, 1.00]
		Electrode	17	16.90	<0.001	0.27	[0.22, 1.00]
		Condition × Electrode	17	0.41	0.98	0.01	[0.00, 100]
	Peak latencies	Condition	1	1.22	0.28	0.05	[0.00, 1.00]
		Electrode	17	7.31	<0.001	0.14	[0.09, 1.00]
		Condition × Electrode	17	0.76	0.74	0.02	[0.00, 100]
